# Exploring the variable importance in random forests under correlations: a general concept applied to donor organ quality in post-transplant survival

**DOI:** 10.1186/s12874-023-02023-2

**Published:** 2023-09-19

**Authors:** Christoph Wies, Robert Miltenberger, Gunter Grieser, Antje Jahn-Eimermacher

**Affiliations:** 1 Department of Mathematics and Natural Sciences, Darmstadt University of Applied Sciences, Schöfferstraße 3, Darmstadt, 64295 Germany; 2 Digital Biomarkers for Oncology, German Cancer Research Center (DKFZ), Im Neuenheimer Feld 223, Heidelberg, 69120, Germany; 3 Medical Facility, University Heidelberg, Im Neuenheimer Feld 672, Heidelberg, 69120 Germany; 4 Department of Computer Science, Darmstadt University of Applied Sciences, Schöfferstraße 3, Darmstadt, 64295 Germany

**Keywords:** Random forest, Permutation variable importance, Resampling test, Kidney transplantation

## Abstract

Random Forests are a powerful and frequently applied Machine Learning tool. The permutation variable importance (VIMP) has been proposed to improve the explainability of such a pure prediction model. It describes the expected increase in prediction error after randomly permuting a variable and disturbing its association with the outcome. However, VIMPs measure a variable’s marginal influence only, that can make its interpretation difficult or even misleading. In the present work we address the general need for improving the explainability of prediction models by exploring VIMPs in the presence of correlated variables. In particular, we propose to use a variable’s residual information for investigating if its permutation importance partially or totally originates from correlated predictors. Hypotheses tests are derived by a resampling algorithm that can further support results by providing test decisions and *p*-values. In simulation studies we show that the proposed test controls type I error rates. When applying the methods to a Random Forest analysis of post-transplant survival after kidney transplantation, the importance of kidney donor quality for predicting post-transplant survival is shown to be high. However, the transplant allocation policy introduces correlations with other well-known predictors, which raises the concern that the importance of kidney donor quality may simply originate from these predictors. By using the proposed method, this concern is addressed and it is demonstrated that kidney donor quality plays an important role in post-transplant survival, regardless of correlations with other predictors.

**Supplementary Information:**

The online version contains supplementary material available at 10.1186/s12874-023-02023-2.

## Introduction

Prediction models are of large interest in medical research. They play, for example, an important role in the US process for allocating donor kidneys to patients due to a severe shortage of kidneys available for transplantation [1]. Next to regression models that are implemented within that allocation process [2, 3], a growing interest can be observed in machine learning methods for investigating important predictors for the post-kidney transplant survival [4, 5]. There is a conflicting debate on how predicting graft survival in kidney transplantation benefits from using machine learning methods [6–8]. Whereas Ravindhran et al. [6] showed that machine learning often predicts outcomes more accurately than regression, Bae et al. [7] argued that the reason could simply be that many machine learning models are trained with more predictors. When training a regression and machine learning model on different kidney transplant outcomes each with the same set of predictors, they could no longer find any relevant differences in prediction performance. As a consequence they argue in favor of regression models because of their explainability.

### Random forests and permutation variable importance

Our research contributes to exploring the explainability of the most commonly applied machine learning methods, the Random Forests. Random Forests were originally introduced by Breiman [9] for regression and classification and have been extended to Random Survival Forests for the analysis of right-censored survival data [10]. Important steps of the algorithm are randomly drawing bootstrap samples and randomly selecting a subset of variables that define the candidates for splitting at each node. These random components decorrelate the trees and thus improve the prediction performance, which is derived from the ensemble of trees. Random Forests operate nonparametrically and are pure prediction models. In contrast to regression models, that provide an estimate of the regression surface, explainability is a concern in Random Forests as well as other pure prediction models [11]. Importance measures have been proposed to describe the contribution of explanatory variables to prediction and thus to connect prediction with the assignment of relevance to individual predictors [9, 12–14]. Efron [15] defined this connection as *attribution*. We here consider the permutation variable importance (VIMP), that is frequently applied as a tool to make Random Forests more explainable [16]. VIMPs measure the importance of a variable as the increase in out-of-bag prediction error that would result from a decorrelation of the outcome and the particular variable by random permutation.

### Limitations of permutation variable importance and proposed solutions

The permutation variable importance measure can be of limited use when information is shared by several variables. Amongst others, Gregorutti et al. [17], Debeer et al. [18] and Efron [15] have shown that correlations and other dependencies between variables affect the VIMPs and can make their interpretation difficult. VIMPs measure a variable’s marginal influence and can suggest a high importance for prediction although it is only low when conditioning the corresponding variable’s information on other features. They can also suggest a low importance when prediction can be derived from different combinations of features as Efron has illustrated in a microarray study of prostate cancer. As a consequence, different methods for improving the explainability of Random Forests by extending the concept of variable importance to *conditional variable importance* have been proposed. Among them Watson and Wright [19] derived a statistical test for the contribution of a set of features to prediction accuracy, conditional on some other pre-selected features [19]. Using the concept of knock-off variables [20], this test investigates effects on the model’s loss function and thus does not refer to any particular statistical model or method. Its benefit in generalizability limits at the same time its usefulness for our purposes because test results do not explicitly refer to VIMPs. For Random Forests, Strobl and others proposed the conditional permutation importance, an approach to better reflect the true importance of each considered feature [18, 21]. The conditional permutation importance describes the conditional influence of a feature by permuting a predictor within strata of other predictors that are correlated with that predictor. Their method of conditional permutation importance is appealing, but also has some limitations: First, it relies on particular implementations of Random Forests, that do not include the *ranger* implementation that is often used for its computational speed [22]. Furthermore, it requires a pre-selection of correlated predictors for building the strata, that is based on *p*-values and thus might depend on sample size. Finally, the concept of conditional permutation importance does not contribute a statistical test for the decrease in importance by correlations, that however could facilitate their interpretation.

### Objective

In this paper, we further contribute to a better understanding of VIMPs. In particular, we address the question whether or not the prediction importance of some selected feature is partially or totally caused by related features. Our methods focus on a single selected feature for which its contribution to prediction is of particular interest and needs explanation. Investigating the role of kidney quality on transplant outcome is an example for such a situation and has motivated this research. For that, we explore the importance of the variable’s residual information and compare it to its marginal importance. Additionally we provide a statistical test for this comparison. The algorithm does not rely on a particular Random Forest implementation. In our accompanying R package we use the *ranger* implementation for its computational speed. We illustrate the derived methods by exploring the importance of kidney quality to post-transplant survival in the presence of many correlated predictors. The analysis is based on about $60\,000$ patient data registered in the transplant information database of the United Network for Organ Sharing (UNOS).

## Motivating example

**Fig. 1:**
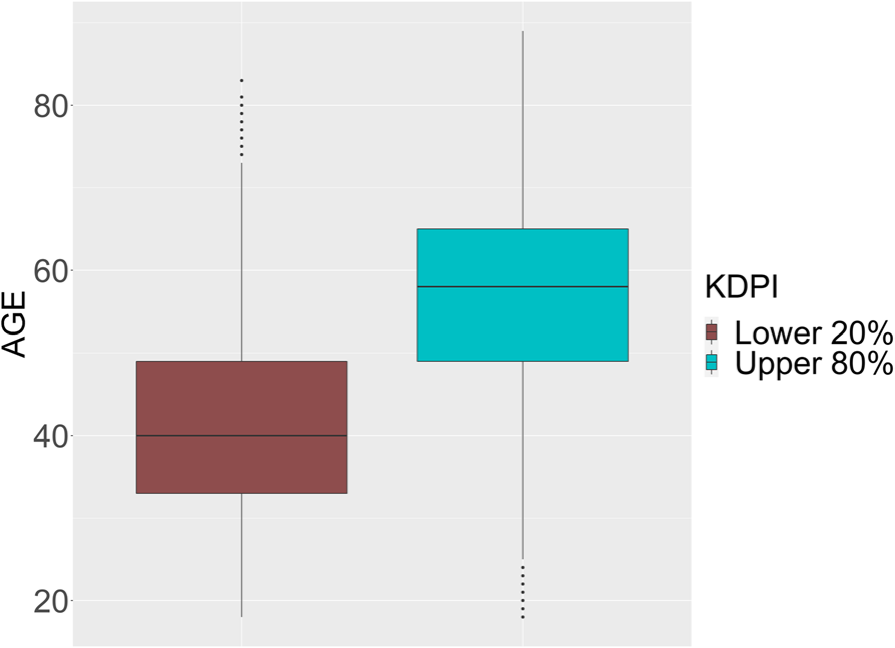
Boxplot of recipient’s age grouped by KDPI values for the patients registered by the United Network of Organ Sharing (UNOS). A detailed description of the population is given in Application section

This work has been motivated by a Random Forest analysis of post-transplant survival following kidney transplantation. Organ transplantation can be an effective therapy for patients suffering from end-stage kidney failure, but there is a severe shortage of donor organs in many countries. Still, many kidneys being available for transplantation are discarded with a median of 16 discarded graft offers per patient [23]. The major reason for discarding is donor quality [23]. To investigate if discarding organs of lower quality is justified by a particular poor prediction, the importance of donor quality for disease-free survival has been investigated in a Random Survival Forest analysis. In fact, the Kidney Donor Profile Index (KDPI) that captures donor quality shows the second highest importance for prediction (see Application section). However, whether organ quality really drives the prediction is unclear, because organ quality is correlated with important other predictors such as age or diabetes disease of the recipient: Transplants of higher quality are offered to patients with a good prognosis, which follows from the American kidney allocation rules. Data of the UNOS [24] with about $60\,000$ kidney transplant observations show that correlation. Figure 1 shows for example the correlation between recipient’s age and donor organ quality. Motivated by the obvious confounding effects we look for a decision criterion, whether the estimated importance of KDPI for prediction is totally or partially driven by correlated variables such as recipient’s age, that are obviously strong predictors for post-transplant survival.

## Methods

### Notations

We consider a situation where the goal is to predict an outcome *Y* by the realization of a set of random variables $X=(X_1,\dots ,X_p)$. The random variables $X_i$ potentially have dependencies to *Y* as well as to other $X_{j,j\ne i}$. We define $X_{-i}=(X_1,\dots ,X_{i-1},X_{i+1},\dots ,X_{p})$ as the vector of all random variables except $X_{i}$. For considering VIMPs we need the concept of a permuted variable. We follow the notation of Gregorutti [17] and define $\pi (X_i)$ as a random permutation of $X_i$, that is defined as an i.i.d. replication of $X_i$ but independent to *X* and *Y*. We use the notation $\pi _i(X)$ for the random vector *X* with permuted i-th coordinate, thus $X_i$ is replaced by $\pi (X_i)$. We define the general statistical model as $Y=f(X)+\epsilon$. Prediction can then be defined as an estimate of the functional *f*(*x*) given $X=x$.

### General concept

Consider a loss function $L: \mathbb {R}^2 \rightarrow \mathbb {R}$ and the expected loss $E\left[ L(Y,f(X))\right]$ when explaining *Y* by some model *f*. Following the definition of Breiman [9] the VIMP for some variable of interest $X_i$ is defined as the difference between the expected loss after and before permuting the values of $X_i$

1 $$\begin{aligned} \text {VIMP}(X_i) = E\left[ L(Y,f(\pi _i(X)))\right] - E\left[ L(Y,f(X))\right] \end{aligned}$$

In Random Forests VIMPs can be estimated from the prediction error in out-of-bag data after and before permuting the values of $X_i$. The idea behind the VIMP is, that for a variable that is not associated with the response, permutation will have no influence on the prediction error and thus the estimated VIMP will be close to zero. For a variable associated with *Y*, permutation will destroy this association and thus the prediction error after permuting will be higher than before permuting and thus the estimated VIMP will be positive.

Commonly applied loss functions are mean squared error in regression forests ($L(x,y)=(x-y)^2$) and 0-1-loss in classification forests ($L(x,y)=I(x\ne y)$). In survival forests *y* is unknown for censored observations and C-index [10] or squared error loss after weighting observations [25] are used to estimate the prediction error.

In the following we propose a general concept for investigating if and how the VIMP of a particular variable is driven by correlations with other predictors. The variable of interest is renamed as *Z* and w.l.o.g. $Z=X_p$. The general idea is to investigate

- if the importance decreases when *Z* is decorrelated from $X_1, \dots , X_{p-1}$
- if the decorrelated part of *Z* still contributes to the prediction of *Y*

For that we first derive the residuals of variable Z when predicted by $X_{-p}$ and then explore the permutation importance of these residuals: We select a class of models G that will be used for explaining Z by $X_1, \dots , X_{p-1}$ and define $g:\mathbb {R}^{p-1}\rightarrow \mathbb {R}$ as the best fitting model within *G*:

2 $$\begin{aligned} g = \underset{\tilde{g} \in G}{argmin}\ E\left[ \left( L(Z,\tilde{g}(X_{-p}))\right) \right] \end{aligned}$$

Assuming additive errors we then can describe Z as

3 $$\begin{aligned} Z=g(X_{-p})+\epsilon _Z \end{aligned}$$

In other words, we separate the part of *Z* that can be explained by $X_1, \dots ,X_{p-1}$ ($g(X_{-p})$) from the part that is independent from $X_1, \dots ,X_{p-1}$ ($\epsilon _Z$). Note, that the loss function used here to model *Z* can differ from the loss function used in the Random Forest analysis of *Y*. The choice of the loss function and class G will be based on the distribution of $(X_1,\dots ,X_{p-1},Z)$.

In a second step, we derive the permutation variable importance $\text {VIMP}(\epsilon _Z)$ of $\epsilon _Z$ from a Random Forest with the independent variables $X_1,\dots , X_{p-1},\epsilon _Z$. To keep in mind that $\text {VIMP}(\epsilon _Z)$ originates from an adjusted model having $X_1,\dots ,X_{p-1},\epsilon _Z$ as predictors, we define $\text {VIMP}_\text {A}(Z)$ as the VIMP of *Z* derived from the original model and $\text {VIMP}_\text {B}(\epsilon _Z)$ as the VIMP of $\epsilon _Z$ derived from the adjusted model.

Exploring $\text {VIMP}_\text {B}(\epsilon _Z)$ compared to $\text {VIMP}_\text {A}(Z)$ will then show whether or not the importance of *Z* partially or totally originates from correlated predictors: A decrease in $\text {VIMP}_\text {B}(\epsilon _Z)$ compared to $\text {VIMP}_\text {A}(Z)$ ($\text {VIMP}_\text {B}(\epsilon _Z) < \text {VIMP}_\text {A}(Z)$) means that the importance of *Z* for predicting *Y* is at least partially caused by information in *Z* that can by explained by other predictors $X_1, \dots , X_{p-1}$. In other words, the importance of variable *Z* borrows importance that originates from other variables. Furthermore, a $\text {VIMP}_\text {B}(\epsilon _Z)$ larger than 0 ($\text {VIMP}_\text {B}(\epsilon _Z)>0$) means that information in *Z* that can not by explained by the other predictors $X_1, \dots , X_{p-1}$ still contributes to predicting *Y*.

For additive models and squared error loss the relations between $\text {VIMP}_\text {A}(Z)$ and $\text {VIMP}_\text {B}(\epsilon _Z)$ are analytically tractable as shown in Appendix A. However, for the more relevant non-additive models that motivate a Random Forest analysis this relation gets lost. Still, exploring differences between $\text {VIMP}_\text {A}(Z)$ and $\text {VIMP}_\text {B}$ can provide useful insight into variable importance which will be illustrated in the application.

As highlighted by one of the reviewers, avoiding overfitting when modeling *g* is of high importance as otherwise residuals will be artificially small and could suggest an artificially small $\text {VIMP}_\text {B}(\epsilon _Z)$. Therefore, we recommend to choose a model class *G* for fitting *g* that addresses overfitting. Simple regression models can be useful in low-dimensional problems whereas ridge regression will be preferable in higher dimensions, where overfitting is reduced by adding a L2-penalty term to the loss function. Also, ensemble methods such as Random Forests could be useful, that reduce the model’s variance by means of bagging. We will discuss a further approach in Discussion section, that we did not yet investigated so far but will be the scope of future research.

In contrast to overfitting, in a misspecified model *Z* can not properly be predicted by $X_{-p}$, that could result in only small differences between $\text {VIMP}_\text {A}(Z)$ and $\text {VIMP}_\text {B}(\epsilon _Z)$ and could underestimate the impact of correlated predictors on the importance of *Z*.

### Statistical test

We now derive statistical tests for the two hypotheses of particular interest. These are:

$$\begin{aligned} \begin{array}{lcl} H_0^{(1)}\ : \, \text {VIMP}_\text {A}(Z)=\text {VIMP}_\text {B}(\epsilon _Z) &{}\text {vs.}&{} H_1^{(1)}\ : \, \text {VIMP}_\text {A}(Z)> \text {VIMP}_\text {B}(\epsilon _Z)\\ H_0^{(2)}\ : \, \text {VIMP}_\text {B}(\epsilon _Z)=0 &{}\text {vs.}&{} H_1^{(2)}\ : \, \text {VIMP}_\text {B}(\epsilon _Z)>0 \end{array} \end{aligned}$$

We define the alternative hypotheses one-sided because residual information will in general not be more important than full information (that can be shown for the special case of additive models, see Appendix A, Eq. (12)) and because of $\text {VIMP}_\text {B}(\epsilon _Z) \ge 0$. The null hypothesis $H_0^{(1)}$ describes the case that the part of *Z* that can be explained by $X_{-p}$ does not improve its variable importance. Thus, $\text {VIMP}_\text {A}(Z)$ describes the actual importance of this variable. In contrast, the alternative hypothesis $H_1^{(1)}$ describes the case that importance does also originate from shared information with other predictors. The null hypothesis $H_0^{(2)}$ describes the case that the part of *Z* that can’t be explained by $X_{-p}$ is of no importance for the prediction.

For deriving the test decision we assume learning samples $(x_i,y_i), \ i=1,\dots ,n$, that are realizations of independently distributed random variables with same distribution as (*X*, *Y*). Using these data, we first estimate the distribution of $\text {VIMP}_\text {B}(\epsilon _Z)$ by resampling: We independently draw *m* random samples from our learning sample, each containing $63.2\%$ of all *n* observations. For each sample we derive $\epsilon _{Z}$ as the vector of residuals of the best fit of *Z* by $X_{-p}$ within model class *G*. Using the *m* random samples with their residual vectors $\epsilon _{Z}$ we compute *m* Random Forests for predicting *Y* by $X_{-p}$ and $\epsilon _{Z}$. With the empirical results for $\text {VIMP}_\text {B}(\epsilon _Z)$ we estimate the corresponding density $d_{\text {VIMP}_\text {B}(\epsilon _Z)}$.

We now compare the observed $\text {VIMP}_\text {A}(Z)$ to this density. The null hypothesis $H_0^{(1)}$ that $\text {VIMP}_\text {A}(Z)$ equals $\text {VIMP}_\text {B}(\epsilon _Z)$ is rejected if $\text {VIMP}_\text {A}(Z)$ exceeds the empirical $(1-\alpha )$-quantile of $d_{\text {VIMP}_\text {B}(\epsilon _Z)}$.

In a second step we compare this density to the value of 0. The null hypothesis $H_0^{(2)}$ is rejected if the $\alpha$-quantile of $d_{\text {VIMP}_\text {B}(\epsilon _Z)}$ exceeds 0.

**Figure:**
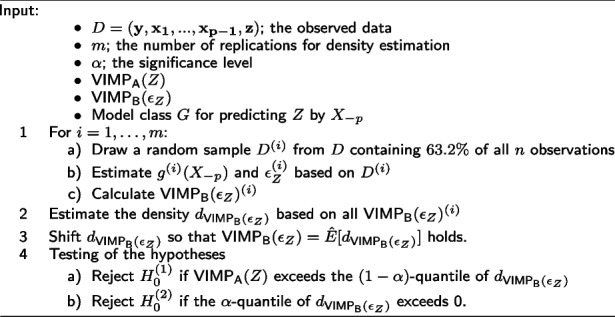
**Algorithm 1** Resampling test

In step 1a) of the Algorithm 1 we use subsamples without replacement instead of the commonly used bootstrap samples with replacement. Bootstrapping results in duplicates during the sample, which causes problems: Bootstrap samples are already drawn within the Random Forest algorithm defining both the forests trainings data as well as the out of bag data for evaluating the corresponding prediction error. Duplicates in the learning sample can therefore cause a single observation to be part of both the training and the out-of-bag sample. This is contradictory to the concept of out-of-sample data.

For subsampling, we apply the 0.632-rule, which means drawing $63.2\%$ of all samples without replacement, to let the probability for each observation to be drawn being the same as the probability to be included in a bootstrap sample of size *n* [26].

For $d_{\text {VIMP}_\text {B}(\epsilon _Z)}$ we use the empirical density with a shift to correct for finite sample sizes. The empirical distribution of $\text {VIMP}_\text {B}(\epsilon _Z)$ is derived from subsamples of size 0.632*n* and we have to consider that finite sample VIMPs only converge with sample size to their asymptotic limits. With $\text {VIMP}^{(n)}(X_i)$ describing the expected VIMP of a variable $X_i$ derived from a sample of size *n* it can be shown that

$$\begin{aligned} \lim _{n\rightarrow \infty }\text {VIMP}^{(n)}(X_i) = \text {VIMP}(X_i). \end{aligned}$$

The proof that relies on some mild assumptions is following ideas of Ishwaran and Lu [27] and is given in Appendix B.

For this reason within Algorithm 1, $E\left[ d_{\text {VIMP}_\text {B}(\epsilon _Z)}\right]$ might systematically differ from full sample $E\left[ \text {VIMP}_\text {B}(\epsilon _Z)\right]$ and thus also from $E\left[ \text {VIMP}_\text {A}(Z)\right]$ even under $H_0$. To correct for this difference, we shift the empirical density $d_{\text {VIMP}_\text {B}(\epsilon _Z)}$ in step 3 of the algorithm, so that $E\left[ d_{\text {VIMP}_\text {B}(\epsilon _Z)}\right] = \text {VIMP}_\text {B}(\epsilon _Z)$ and then use this estimate in step 4 of Algorithm 1 to test our hypotheses.

The proposed statistical tests investigate the contribution of a particular variable of interest to prediction. If that variable shows a high importance measured by $\text {VIMP}_\text {A}(Z)$, an investigation of its conditional importance can provide further insight. If that variable shows a small importance $\text {VIMP}_\text {A}(Z)$ only, its further investigation will be of minor interest.

The interplay between $\text {VIMP}_\text {B}(\epsilon _Z)$ and the permutation importance of other variables within the same model can provide interesting insights. If the importance of some variable $X_i$ increases when *Z* is replaced by its residuals $\epsilon _Z$, this indicates that a part of variable’s *Z* importance might originate from variable $X_i$. We will observe this pattern within the data example (Application section).

More difficult is the interpretation in situations where not only one selected variable is of main interest. Applying the proposed method to several variables will for example not answer the question which variable is a better predictor because the results will depend on the presence and degree of correlations with further variables. For the same reason, if for two variables $H_0^{(1)}$ can be rejected while $H_0^{(2)}$ is accepted, the reason could either be that both variables share the same information or that the two variables carry very different information each of which is shared with third variables.

### Implementation

We implemented our methods as a R package called *RVIMP*^1^ which is an abbreviation for ResidualVIMP as our method relies on the density $d_{\text {VIMP}_\text {B}(\epsilon _Z)}$ of VIMPs calculated for residuals.

For Random Forest analysis within our algorithm we apply the package *ranger* [22] and benefit from its fast implementation.

The most computational intensive part of our test is step 1 in Algorithm 1. Especially for survival data this step is time-consuming. So the replication parameter *m* is crucial for computational time. In our simulations we used $m=100$, which showed satisfactory results.

Besides the test procedure the package provides a visualization of the test result and a comparison between $\text {VIMP}_\text {A}$ and $\text {VIMP}_\text {B}$ for each variable $X_i$.

## Simulation study

In the following we will evaluate the proposed test procedures by the use of simulations.

*Objectives:* The aim of this simulation study is to figure out, whether our test is able to control the $\alpha$-error rates and how the power depends on sample size and the degree of correlations between the variables and to the outcome. Empirical type I error and power are investigated both for regression and classification forest analyses.

Simulation Design A:

For investigating empirical type I error and power for a regression forest analysis data are simulated that follow the linear model

4 $$\begin{aligned} Y = b_1X_1+b_2X_2+b_3X_3+b_4X_4+b_5X_5+b_6Z + \epsilon _1 \end{aligned}$$

with $\epsilon _1\sim N(0,\sigma _1^2)$. Realizations of $X=(X_1,X_2, \dots , X_5)$ are simulated as i.i.d. random samples from a multivariate normal distribution with $E(X_i)=0$ and $Var(X_i)=1$ for $i=1, \dots , 5$. Correlations are specified as $C(X_1,X_2)=c>0$ and $C(X_1,X_i)=C(X_2,X_i)=C(X_i,X_j)=0$ for $i,j > 2$ and $i\ne j$.

The variable of interest *Z* is defined conditional on $X_1$ and $X_2$ by

$$\begin{aligned} Z = 0.5X_1+0.5X_2 + \epsilon _2 \end{aligned}$$

with $\epsilon _2 \sim N(0,\sigma _2^2)$. The parameters $b_1$,...,$b_6$, $\sigma _1^2$, and $\sigma _2^2$ are set to values that induce a correlation *C*(*Y*, *Z*) between Y and Z of 0.3, 0.6 or 0.9 and a semipartial correlation $spC(Y,Z|X_1,\dots , X_5)=C(Y,\epsilon _2)$ that is reduced by 1/3 or 2/3, respectively. For that we chose the regression parameters as $b_1=b_2=0.5$, $b_3=b_6=1$, $b_4=b_5=0$. Details on how to identify $\sigma _1^2$ and $\sigma _2^2$ that result in a particular correlation and semipartial correlation are given in Appendix C, Table A1 together with the identified values. The correlation *c* between $X_1$ and $X_2$ was defined to be equal to *C*(*Y*, *Z*).

Furthermore, a design with no semipartial correlation at all between Y and Z was considered. For this design the model parameters of eq. (4) are set to $b_1=b_2=b_3=1$ and $b_4=b_5=b_6=0$. For each scenario $1\,000$ datasets with each 500, $1\,000$, or $5\,000$ observations are generated, respectively. For each of these datasets we apply the proposed resampling tests to the variable of interest *Z* at a local significance level of $\alpha =0.05$. Thereby, we apply a linear model for *g*. In the considered simulation designs $H_0^{(1)}$ is true if $C(Y,Z)=spC(Y,Z|X_1,\dots , X_5)$ and $H_0^{(2)}$ is true if $spC(Y,Z|X_1,\dots , X_5)=0$. For each null hypothesis and simulation design we derive the empirical rejection rate over the $1\,000$ replications. In simulation designs with $H_0^{(k)}$ being true this provides an estimate of the $\alpha$-error rate. In simulation designs with $H_0^{(k)}$ being false this provides an estimate of the power.

To further investigate $\alpha$-error rates for $H_0^{(1)}$ and $H_0^{(2)}$ we apply our test procedure also to variable $X_4$. For this variable $H_0^{(1)}$ and $H_0^{(2)}$ both are true in each simulation design as $C(Y,X_4)=spC(Y,X_4)=0$. The results for this setup are shown in the Appendix D.a in Table A2.

We perform $1\,000$ replications only as our test procedure is computational intensive. Thus, random variation in the estimates derived from the simulations must be considered when interpreting results.

Simulation Design B:

To investigate the proposed test procedures also for classification forests, we slightly adjusted the simulation design: The dependent variable *Y* was defined as binary with a logistic response function applied to the linear predictor:

$$\begin{aligned} Y\sim B(1,p) \quad \text {with}\ p=(1+\exp (-(b_1X_1+b_2X_2+b_3X_3+b_4X_4+b_5X_5+b_6Z+\epsilon _1)))^{-1} \end{aligned}$$

with $b_1 = b_2 = b_3 = 1$ and $b_4 = b_5 = b_6 = 0$. We use the same distribution of $(X_1, \dots X_5, Z)$ with $c=0.3$, the same distribution of $\epsilon _1$ and $\epsilon _2$ with $\sigma _1^2=4.04$ and $\sigma _2^2=0.26$ and the same choice for *g* as applied in Simulation Design A. We did not vary $\sigma _1$, $\sigma _2$, and *c* because these parameters do not define partial and semipartial correlations anymore in a classification setting. Instead, we applied our proposed test on all 6 variables to investigate different setups with respect to both hypotheses.

Simulation Design C:

Additionally we investigated another simulation design for regression forests with more variables. Details about Simulation Design C are given in Appendix D.b.

### Simulation results

The results when applying the test procedures to variable *Z* in Simulation Design A are shown in Table 1.

**Table 1:** Simulation results (1000 simulated datasets, each with *n* patients) showing empirical rejection probabilities of the proposed resampling tests for a regression Random Forest. Correlations within the simulation model are given as the correlation between *Y* and *Z* (*C*(*Y*, *Z*)) and semipartial correlation between *Y* and *Z* ($spC(Y,Z):=spC(Y,Z|X_1, \dots , X_5)$)

Simulation design		Rejection probability of $\bf H_0^{(1)}$			Rejection probability of $\bf H_0^{(2)}$		
*C*(*Y*, *Z*)	*spC*(*Y*, *Z*)	*n*=500	*n*=1000	*n*=5000	*n*=500	*n*=1000	*n*=5000
0.3	0	0.946	0.995	1.000	0.019	0.027	0.020
0.3	0.1	0.985	0.999	1.000	0.237	0.396	0.937
0.3	0.2	0.538	0.801	0.999	0.775	0.968	1.000
0.6	0.2	1.000	1.000	1.000	0.946	1.000	1.000
0.6	0.4	0.994	1.000	1.000	1.000	1.000	1.000
0.9	0.3	1.000	1.000	1.000	1.000	1.000	1.000
0.9	0.6	0.999	1.000	1.000	1.000	1.000	1.000

When the null hypothesis $H_0^{(2)}$ is true (that is the case when $spC(Y,Z)=0$) it is rejected with a probability between 0.019 and 0.027. These rates suggest that the test keeps the $5\%$ significance level for $H_0^{(2)}$. Results in Appendix D, Table A2 further confirm this result by showing rejection probabilities of $H_0^{(2)}$ between 0.02 and 0.057 when applied to variable $X_4$ for different simulation designs and sample sizes. We do not consider this as a violation of the anticipated $5\%$-error-level because of the quite large standard error of an empirical rate in only 1000 replications (SE=0.007).

The power for rejecting $H_0^{(1)}$ ranges between 0.538 and 1 and depends, as common for statistical tests, on the one hand on the number of observations *n* and on the other hand on the difference between *C*(*Y*, *Z*) and *spC*(*Y*, *Z*). As expected, the power increases with increasing *n* as well as with increasing difference between *C*(*Y*, *Z*) and *spC*(*Y*, *Z*).

The power for rejecting $H_0^{(2)}$ ranges between 0.237 and 1. As expected it increases with *n* and with increasing *spC*(*Y*, *Z*).

The results from Simulation Design B, where we investigated a classification task, are shown in Table 2. The results for the classification task confirm our results of simulation design A: The test holds the type I error rates close to the significance level of $\alpha =5\%$ with rates for $H_0^{(1)}$ between 0.000 and 0.002 and for $H_0^{(2)}$ between 0.045 and 0.074. Again, we consider the slightly increased error rates for $H_0^{(2)}$ as random variations. The power for rejecting $H_0^{(1)}$ ranges between 0.154 and 1.000 and the power for rejecting $H_0^{(2)}$ ranges between 0.776 and 1.000 in Simulation Design B. For both hypotheses it increases as expected with increasing *n*.

**Table 2:** Simulation results (1000 simulated datasets, each with *n* patients) showing empirical rejection probabilities of the proposed resampling tests for a classification Random Forest. For all variables the information is given whether or not $H_0^{(1)}$ and $H_0^{(2)}$ is true. Thereby, $H_0^{(1)}$ is considered to be true for variables that are independent to all variables $X_i$ with non-zero regression coefficient ($X_3, X_4, X_5$) and $H_0^{(2)}$ is considered to be true when the variable’s regression coefficient is zero ($X_4$, $X_5$, *Z*)

Variable			Rejection probability of $\bf H_0^{(1)}$			Rejection probability of $\bf H_0^{(2)}$		
Z=	$\bf H_0^{(1)}=$	$\bf H_0^{(2)}=$	*n*=500	*n*=1000	*n*=5000	*n*=500	*n*=1000	*n*=5000
$X_1$	false	false	0.300	0.571	1.000	0.797	0.969	1.000
$X_2$	false	false	0.303	0.561	0.999	0.776	0.963	1.000
$X_3$	true	false	0.000	0.000	0.000	0.866	0.985	1.000
$X_4$	true	true	0.000	0.000	0.002	0.061	0.058	0.066
$X_5$	true	true	0.000	0.002	0.001	0.063	0.050	0.074
*Z*	false	true	0.154	0.226	0.572	0.045	0.065	0.063

The results for Simulation Design C are shown in Appendix D.b in Table A3.

## Application: Exploring the importance of kidney donor organ quality for post-transplant survival

We applied the proposed methods to the American kidney transplantation data as provided by the *United Network for Organ Sharing* (UNOS) [24]. The methods are used to investigate the importance of donor organ quality (KDPI score) for prediction in the presence of correlations with recipients’ characteristics (see Motivating example section). A Random Survival Forest is fitted to post-transplant survival as outcome variable, defined as the time from transplantation to recipient’s death, graft failure or graft rejection whatever happens first. Data of about $60\,000$ adult patients is used, where every patient has received a single deceased donor kidney while not waiting for further donor organs and not having received any organ transplantation before. Only patients with a transplantation date between 01/01/2015 and 02/01/2020 are considered because the allocation process has changed in 2015.

To investigate the importance of KDPI for prediction and how its importance is driven by correlated predictors, KDPI is included as the variable that combines 10 donor factors together with 28 characteristics that describe the recipient and the transplantation procedure. The selection of the recipient and transplantation characteristics was motivated by the review of Kabore et al. [28]. Next to frequently used variables according to [28] we further included additional variables with a low frequency of missing values. A full list of all variables is shown in the Appendix E.

**Fig. 2:**
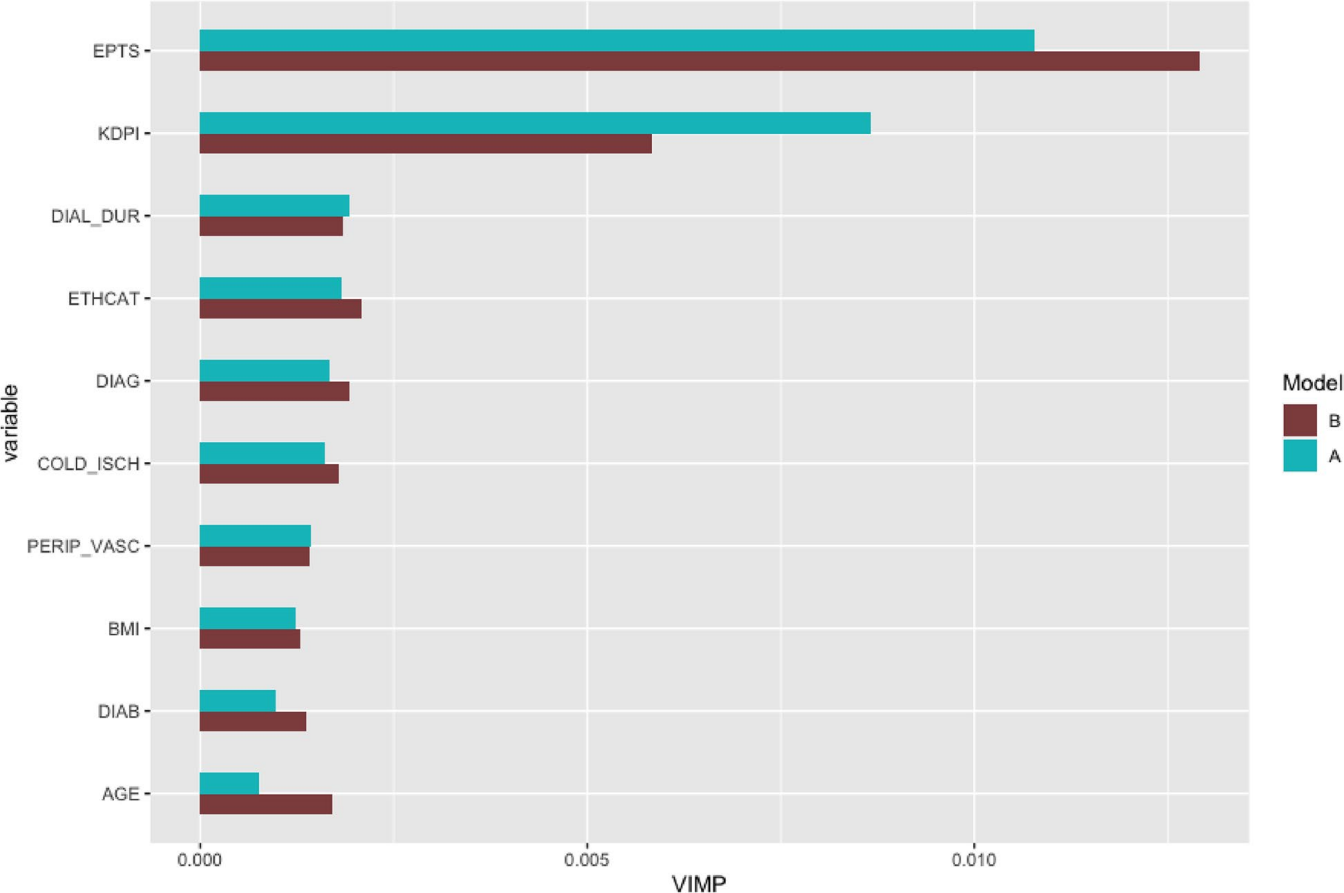
The figure shows the VIMPs of the 10 variables with highest importance in the Random Survival Forest analysis of UNOS data under model description $\text {model}_\text {A}$ as well as the corresponding VIMPS under model description $\text {model}_\text {B}$. Thereby, in $\text {model}_\text {B}$ KDPI was replaced by its residuals whereas all other variables remained unchanged. Thus, $\text {VIMP}_\text {B}(\text {KDPI})$ refers to residual information and all other VIMPs to original variables’ information within the two models

Figure 2 shows the 10 highest VIMPs under model description $\text {model}_\text {A}$ of all 29 variables that were investigated in the Random Survival Forest analysis. Each VIMP is presented together with the corresponding VIMP of $\text {model}_\text {B}$, where *Z*=KDPI is replaced by its residuals $\epsilon _Z$. Thereby, $\epsilon _Z$ is derived from a Random Forest to allow for non-linear and non-additive dependencies between KDPI and the other variables. The VIMPs of all variables are shown in the Appendix F in Fig. A2.

KDPI shows the second largest importance for prediction (VIMP=0.0087). Only the expected post transplantation survival (EPTS) shows a larger importance. EPTS is an aggregated score containing for example the recipients age, thus its high importance is not surprising. The importance of KDPI substantially decreases when considering only its residuals in the Random Survival Forest analysis whereas the importance of EPTS as well as variables contributing to EPTS such as recipient’s age and diabetes increases. This suggests that the importance of KDPI is partially caused by these correlations, that arise from the allocation procedure as described in Motivating example section. However, KDPI remains the second most important predictor besides EPTS.

**Fig. 3:**
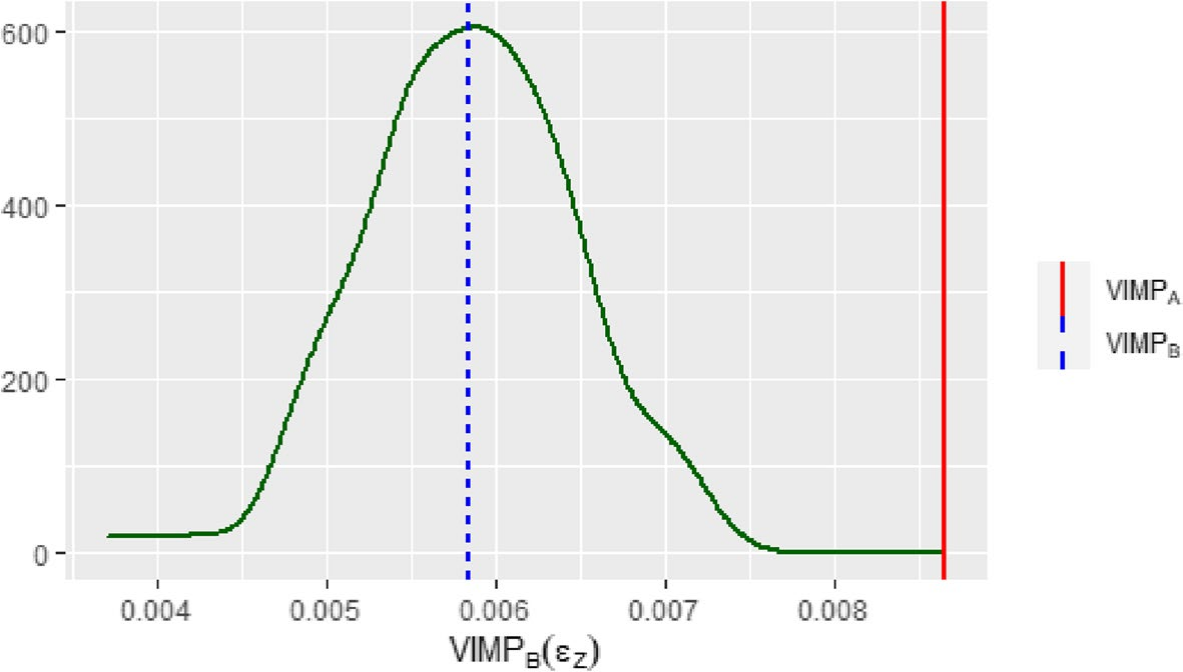
The figure shows the test result for variable KDPI. The observed $\text {VIMP}_\text {A}$ for KDPI (solid vertical line) exceeds the $95\%-$quantile of the estimated density of $\text {VIMP}_\text {B}(\epsilon _Z)$. The dotted vertical line shows the observed $\text {VIMP}_\text {B}(\epsilon _Z)$ for KDPI

The statistical test results are illustrated in Fig. 3. The observed marginal VIMP of 0.0087 exceeds the 95%-quantile of the density $$d_{\text {VIMP}_\text {B}(\epsilon _Z)}$$, which provides a statistically significant test result for $$H_0^{(1)}$$ ($$p<0.001$$). This confirms, that the importance of KDPI is at least partially caused by sharing information with correlated predictors. However, $$H_0^{(2)}$$ can also be rejected ($$p<0.001)$$ indicating still a conditional importance of KDPI. In summary, the test results fit well to the results given in Fig. 2.

## Discussion

In simulated and real data we have demonstrated the usefulness of investigating a variable’s residual information together with statistical tests for the hypotheses that the variable’s importance partially or totally originates from correlated predictors. It can give further insight into a variable’s importance for prediction and thus contributes to the general need for improving the explainability of machine learning results. Given that Random Forests are only one of many options for prediction modeling in organ transplantation [6, 29, 30] and a lack of consensus about the meaning of *variable importance*, there will not be a single answer to the question of explainability and our work contributes one piece of information to that question.

### Issues and recommendations for applications

Our methods refer to applications where a single variable might be of particular interest with respect to its importance for prediction. This must be differentiated from another active field of research on how to use variable importance for variable selection [31–35] possibly based on *p*-values [36]. Our research has been motivated by an investigation of the role of kidney quality for post-transplant survival and this example can give some guidance on how to apply our method and interpret its results. The variable of interest was kidney quality (measured as KDPI) and we could demonstrate that its high importance for prediction partially originates from patient characteristics that are correlated to KDPI due to the allocation policy. However, our results also confirm that irrespective of these correlations kidney quality still is a major predictor for post-transplant survival even if not as high as its VIMP originally would suggest. The latter results might support findings of Bae et al. [37], who question the need for rejecting many kidneys of lower quality in the presence of a severe shortage of donor organs.

Our methods do not rely on a particular implementation of Random Forests, as the algorithm itself is not adapted but is applied to residual information that are derived in a preceding step (see Algorithm 1). This is an advantage towards for example the investigation of Conditional Permutation Importance (CPI) [18, 21] that rely on particular R implementations and are at least currently not compatible with ranger or Python implementations. However, CPIs have the advantage that conditional importance is derived by permuting a variable within strata of correlated variables and therefore do not rely on the specification of some model *g* and its accuracy.

To make the methods easily accessible and facilitate their application, we provide an implementation within the statistical software R. It uses the *ranger* implementation that is helpful in particular when it comes to the computationally challenging permutation tests.

### Limitations and extensions

One limitation of the proposed algorithm is that it cannot clearly identify which variable or group of variables contribute to the importance of the variable of interest. To some extent this question can be explored by investigating how the importance of other variables changes when considering only the residual information of the variable of interest, but this will neither clearly identify nor quantify correlations. Furthermore, it is important to note that neither causal pathways nor the direction of causal effects can be identified with the proposed methods.

Our simulation studies showed that the proposed statistical tests control the type I error. However, it must be considered that the reported empirical error rates have high standard errors as the number of simulations was limited by computational restrictions. For the same reason we could provide simulation results only for low-dimensional settings but not for situations that usually motivate machine learning: large sample sizes with many predictors. This is a common drawback of simulation studies for Random Forests [18, 19, 21].

As Random Forests operate nonparametrically, we could not rely on a particular statistical distribution when deriving the test procedure. Instead, we used resampling techniques. Here, double-bootstrapping had to be circumvented by drawing subsamples of the learning data without replacement as has also been used by Ishwaran and Lu when investigating the variability of VIMPs [27].

As discussed before, a drawback of the proposed method is that it relies on a reasonable choice of model *g* that does not suffer from overfitting. We believe that our algorithm could further be improved towards that direction by training *g* under cross-validation and deriving the residuals $\epsilon _Z$ from the leave-out folds only. A further alternative could be the use of knock-off variables [20] instead of residual information, that also relies on much less assumptions. Both will be investigated in future work.

### Conclusion

Random Forest analyses are often accomplished by a description of the variable’s importance for prediction, that usually is defined as permutation importance. However, interpretability of VIMPs can be disturbed when predictors are correlated as has been highlighted by different researchers [15, 17, 18]. Our methods can improve its interpretation for single variables for which the contribution to prediction is of particular interest and needs explanation. Investigating the role of kidney quality on transplant outcome is only one example for such a situation and has motivated this research.

Conditional importance has also been investigated by Strobl and others [18, 21]. Compared to them, our approach does not rely on particular software implementations and is enhanced by statistical test results. This however comes at the price of being less flexible with respect to the pattern of correlations between predictors.

### Supplementary Information

**Additional file 1.**

## Data Availability

The data reported here have been supplied by the United Network for Organ Sharing as the contractor for the Organ Procurement and Transplantation Network (data request number DATA0005808). The interpretation and reporting of these data are the responsibility of the authors and in no way should be seen as an official policy of or interpretation by the OPTN or the U.S. Government. Based on OPTN data as of June 20, 2020. Data can be requested at: https://optn.transplant.hrsa.gov/data/view-data-reports/request-data/ [24].

## Abbreviations

EPTS: Expected Post Transplantation Survival
KDPI: Kidney Donor Profile Index
UNOS: United Network for Organ Sharing
VIMP: Permutation Variable Importance

## Footnotes

1 https://github.com/romilt/RVIMP
