# Supplementary material for: Exploring the variable importance in random forests under correlations: a general concept applied to donor organ quality in post-transplant survival
Source: BMC Med Res Methodol. 2023 Sep 19;23:209. doi: 10.1186/s12874-023-02023-2 (PMC10507897; doi:10.1186/s12874-023-02023-2)
Supplement: Supplementary file 1 — Additional file 1. [file 12874_2023_2023_MOESM1_ESM.pdf]

Appendix

A) Variable and its residual importance in additive models

For regression forests and squared error loss the permutation variable importance is given by:

$$\text{VIMP}(X_i) = E\left[(Y - f(\pi_i(X)))^2\right] - E\left[(Y - f(X))^2\right]. \tag{5}$$

When further assuming the underlying model to be additive and w.l.o.g. the intercept to be 0:

$$Y = f_1(X_1) + \cdots + f_p(X_p) + \epsilon \tag{6}$$

this formula further simplifies:

$$\begin{aligned}
\text{VIMP}(X_i) &= E\left[(Y - f(\pi_i(X)))^2\right] - E\left[(Y - f(X))^2\right] \\
&= E\left[(f(X) - f(\pi_i(X)))^2\right] + 2E\left[(Y - f(X)) \cdot (f(X) - f(\pi_i(X)))\right] \\
&= E\left[(f_i(X_i) - f_i(\pi(X_i)))^2\right] + 2E\left[\epsilon\right] E\left[f(X) - f(\pi_i(X))\right] \\
&= E\left[(f_i(X_i) - E[f_i(X_i)])^2\right] + E\left[(f_i(\pi(X_i)) - E[f_i(\pi(X_i))])^2\right] \\
&\quad + 2E\left[(f_i(X_i) - E[f_i(X_i)]) \cdot (f_i(\pi(X_i)) - E[f_i(\pi(X_i))])\right] \\
&= 2 \cdot Var(f_i(X_i))
\end{aligned} \tag{7}$$

In the derivation it was used that $f_i(X_i)$ and $f_i(\pi(X_i))$ are independent but still have the same distribution and thus the same expected value. In particular, it can be seen that the VIMP can also be expressed as

$$\text{VIMP}(X_i) = E\left[(f_i(X_i) - f_i(\pi(X_i)))^2\right]. \tag{8}$$

With renaming the variable of interest as $Z = X_p$ the model from equation (6) which we call model_A is described as:

$$Y = f_1(X_1) + f_2(X_2) + \cdots + f_{p-1}(X_{p-1}) + f_p(Z) + \epsilon \tag{model_A}$$

We now define the model where shared information is removed from Z, which we call model_B. For that, we separate the part of Z that can be explained by $X_1, \ldots, X_{p-1}$ from the part that is independent from $X_1, \ldots, X_{p-1}$ by defining $g : \mathbb{R}^{p-1} \to \mathbb{R}$ as described in equation (3) and apply mean squared error as loss function:

$$g = \underset{\tilde{g} \in G}{argmin}\ E\left[(Z - \tilde{g}(X_{-p}))^2\right] \Rightarrow\ Z = g(X_{-p}) + \epsilon_Z \tag{9}$$

The two parts of model (9), namely $g(X_{-p})$ and ϵ_Z, are independent and describe the explained and unexplained part of Z, respectively.

We now assume that f_p is additive in $g(X_{-p})$ and ϵ_Z:

$$f_p(Z) = f_p(g(X_{-p}) + \epsilon_Z) = h_2(g(X_{-p})) + h_1(\epsilon_Z) \tag{10}$$

where $h_1(\epsilon_Z)$ and $h_2(g(X_{-p}))$ are functions of independent random variables and are therefore also independent. The assumption on additivity of f_p is fairly reasonable considering the flexibility of additive model components.
Inserting equation (10) into equation (model$_\mathrm{A}$) leads to an alternative model description:

$$\begin{aligned} Y &= f_1(X_1) + \cdots + f_{p-1}(X_{p-1}) + f_p(Z) + \epsilon \\ &= f_1(X_1) + \cdots + f_{p-1}(X_{p-1}) + h_2(g(X_{-p})) + h_1(\epsilon_Z) + \epsilon \\ &= \tilde{f}(X_1, \ldots, X_{p-1}, \epsilon_Z) + \epsilon \qquad \text{(model}_\mathrm{B}\text{)} \end{aligned}$$

Now, $\mathrm{VIMP}_\mathrm{A}(\cdot)$ describes the VIMPs of model$_\mathrm{A}$ as defined in equation (5) and is defined for $X_1, \ldots, X_{p-1}$ and Z. In contrast, $\mathrm{VIMP}_\mathrm{B}(\cdot)$ refers to model$_\mathrm{B}$ and is defined for $X_1, \ldots, X_{p-1}$ and ϵ_Z:

$$\begin{aligned} \mathrm{VIMP}_\mathrm{B}(X_i) &= E\left[(Y - \tilde{f}(\pi_i(X_1, ..., X_{p-1}, \epsilon_Z)))^2\right] - E\left[(Y - \tilde{f}(X_1, \ldots, X_{p-1}, \epsilon_Z))^2\right] \\ \mathrm{VIMP}_\mathrm{B}(\epsilon_Z) &= E\left[(h_1(\epsilon_Z) - h_1(\pi(\epsilon_Z)))^2\right] \end{aligned}$$

For the last equation we used the general result on VIMPs given in (8).
We now show that $\mathrm{VIMP}_\mathrm{B}(\epsilon_Z)$ can never exceed $\mathrm{VIMP}_\mathrm{A}(Z)$ and identify the situation where equality holds.

$$\begin{aligned} \mathrm{VIMP}_\mathrm{A}(Z) &= E\left[(f_p(Z) - f_p(\pi(Z))^2\right] \\ &= E\left[(h_2(g(X_{-p})) + h_1(\epsilon_Z) - h_2(\pi(g(X_{-p}))) - h_1(\pi(\epsilon_Z)))^2\right] \\ &= E\left[(h_1(\epsilon_Z) - h_1(\pi(\epsilon_Z)))^2\right] + E\left[(h_2(g(X_{-p})) - h_2(\pi(g(X_{-p}))))^2\right] \\ &\quad +2E\left[(h_2(g(X_{-p})) - h_2(\pi(g(X_{-p})))) \cdot (h_1(\epsilon_Z) - h_1(\pi(\epsilon_Z)))\right] \\ &= \mathrm{VIMP}_\mathrm{B}(\epsilon_Z) + E\left[(h_2(g(X_{-p})) - h_2(\pi(g((X_{-p}))))^2\right] + 0 \qquad (11) \\ &\geq \mathrm{VIMP}_\mathrm{B}(\epsilon_Z) \qquad (12) \end{aligned}$$

The last term in equation (11) is 0 because $h_1(\epsilon_Z)$ and $h_2(g(X_{-p}))$ are uncorrelated and $E\left[h_1(\epsilon_Z)\right] = E\left[h_1(\pi(\epsilon_Z))\right]$. The term $E\left[(h_2(g(X_{-p})) - h_2(\pi(g((X_{-p}))))^2\right]$ in (11) can be considered as a hypothetical VIMP of $g(X_{-p})$, that would result if this explained part of Z would have been included as a separate variable into the model. Finally, we get:

$$\mathrm{VIMP}_\mathrm{B}(\epsilon_Z) = \mathrm{VIMP}_\mathrm{A}(Z) \Leftrightarrow E\left[(h_2(g(X_{-p})) - h_2(\pi(g((X_{-p}))))^2\right] = 0 \qquad (13)$$

Relation (13) describes that $\mathrm{VIMP}_\mathrm{A}(Z)$ and $\mathrm{VIMP}_\mathrm{B}(\epsilon_Z)$ are of same size if and only if the explained part of Z, i.e. $g(X_{-p})$, would not improve the loss of the Random Forest. In other words, the part of Z that can be explained by $X_1, \ldots, X_{p-1}$ (within the model class G) does not explain Y.

B) Asymptotics of finite sample VIMPs

In this section we show, that the finite sample VIMP estimated from a Random Forest converges with sample size to the VIMP as defined in (5). This convergence is

the motivation why the density $d_{\mathrm{VIMP_B}(\epsilon_Z)}$ estimated from resampled $\mathrm{VIMP_B}(\epsilon_Z)$ has to be adjusted: $\mathrm{VIMP_B}(\epsilon_Z)$ as derived from a sample of size $0.632n$ might show stronger deviations from its asymptotic limit as $\mathrm{VIMP_B}(\epsilon_Z)$ is derived from the full sample and thus might systematically differ from the full sample $\mathrm{VIMP_B}(\epsilon_Z)$.
The proof follows ideas of Ishwaran and Lu [27] and relies on some assumptions as described in the following. For a discussion, why these assumption can be considered as fairly reasonable, we refer to [27].
Let $\mathrm{VIMP}^{(n)}(X_i)$ be the VIMP of X_i estimated from a learning sample of n independent observations with same distribution as (X,Y). We again assume $Y = f(X)+\epsilon$ and define $f^{(n)}(X)$ as the predictor of Y derived from a Random Forest analysis of the learning sample. Then

$$\begin{aligned}
\mathrm{VIMP}^{(n)}(X_i) &= E\Big[\Big(Y - f^{(n)}(\pi_i(X))\Big)^2\Big] - E\Big[\Big(Y - f^{(n)}(X)\Big)^2\Big] \\
&= E\Big[\Big(f^{(n)}(\pi_i(X)) - Y + Y - f^{(n)}(X)\Big)^2 \\
&\qquad +2\Big(Y - f^{(n)}(\pi_i(X))\Big)\Big(Y - f^{(n)}(X)\Big) - 2\Big(Y - f^{(n)}(X)\Big)^2\Big] \\
&= E\Big[\Big(f^{(n)}(\pi_i(X)) - f^{(n)}(X)\Big)^2 - 2\Big(Y - f^{(n)}(X)\Big)^2 \\
&\qquad -2\Big(f^{(n)}(\pi_i(X)) - f^{(n)}(X) - Y + f^{(n)}(X)\Big)\Big(Y - f^{(n)}(X)\Big)\Big] \\
&= E\Big[\Big(f^{(n)}(\pi_i(X)) - f^{(n)}(X)\Big)^2 \\
&\qquad -2\Big(Y - f^{(n)}(X)\Big)\Big(Y - f^{(n)}(X) + f^{(n)}(\pi_i(X)) - f^{(n)}(X) - Y + f^{(n)}(X)\Big)\Big] \\
&= E\Big[\Big(f^{(n)}(\pi_i(X)) - f^{(n)}(X)\Big)^2 - 2\Big(Y - f^{(n)}(X)\Big)\Big(f^{(n)}(\pi_i(X)) - f^{(n)}(X)\Big)\Big] \\
&= E\Big[\Big(f^{(n)}(\pi_i(X)) - f^{(n)}(X)\Big)^2 - 2\Big(f(X) - f^{(n)}(X)\Big)\Big(f^{(n)}(\pi_i(X)) - f^{(n)}(X)\Big) \\
&\qquad -2\epsilon\Big(f^{(n)}(\pi_i(X)) - f^{(n)}(X)\Big)\Big] \\
&= E\Big[\Big(f^{(n)}(\pi_i(X)) - f^{(n)}(X)\Big)^2\Big] + 2A_n - 0 \\
&= E\Big[\Big(f^{(n)}(\pi_i(X)) + f(\pi_i(X)) - f(\pi_i(X)) - f^{(n)}(X) + f(X) - f(X)\Big)^2\Big] + 2A_n \\
&= E\Big[\Big(f(\pi_i(X)) - f(X)\Big)^2 + \Big(f^{(n)}(\pi_i(X)) - f(\pi_i(X)) - f^{(n)}(X) + f(X)\Big)^2\Big] \\
&\quad +2E\Big[\Big(f(\pi_i(X)) - f(X)\Big)\Big(f^{(n)}(\pi_i(X)) - f(\pi_i(X)) - f^{(n)}(X) + f(X)\Big)\Big] \\
&\quad +2A_n
\end{aligned} \tag{14}$$

where A_n is defined as $E\Big[\Big(f^{(n)}(X) - f(X)\Big)\Big(f^{(n)}(\pi_i(X)) - f^{(n)}(X)\Big)\Big]$. Now define the residuals of the forest when applied to X and its permutated variant as $r_n(X)$ and $r_n(\pi_i(X))$, respectively:

$$r_n(X) := f^{(n)}(X) - f(X) \qquad r_n(\pi_i(X)) := f^{(n)}(\pi_i(X)) - f(\pi_i(X))$$

With an exchangeability assumption on $(r_n(X), r_n(\pi_i(X)))$ it follows that

$$E\Big[\Big(f(\pi_i(X)) - f(X)\Big)\Big(r_n(\pi_i(X)) - r_n(X)\Big)\Big] = 0$$

and thus the second expectation in (14) is 0. In summary,

$$\begin{aligned}\text{VIMP}^{(n)}(X_i) &= E\Big[\Big(f(\pi_i(X)) - f(X)\Big)^2 + \Big(r_n(\pi_i(X)) - r_n(X)\Big)^2\Big] + 2A_n \\ &= E\Big[\Big(f(\pi_i(X)) - f(X)\Big)^2\Big] + 2\sigma_n^2(1-\rho_n) + 2A_n\end{aligned}$$

with $\sigma_n^2 := Var(r_n(\pi_i(X)) = Var(r_n(X))$ and $\rho_n := C(r_n(\pi_i(X)), r_n(X))$. This relies on a further assumption that $f^{(n)}$ is L_2-consistent for f. With this assumption it can also be shown, that A_n converges to 0:

$$\begin{aligned}A_n &= E\Big[\Big(f^{(n)}(X) - f(X)\Big)\Big(f^{(n)}(\pi_i(X)) - f^{(n)}(X)\Big)\Big] \\ &\leq \sqrt{E[(f^{(n)}(X) - f(X))^2]}\sqrt{E[(f^{(n)}(\pi_i(X)) - f^{(n)}(X))^2]}\end{aligned}$$

as $E[(f^{(n)}(\pi_i(X)) - f^{(n)}(X))^2]$ can be shown to be bounded [27] and $E[(f^{(n)}(X) - f(X))^2]$ converges to 0. With L_2-consistency of $f^{(n)}$ it follows that σ_n^2 also converges to 0 and finally

$$\lim_{n\to\infty} \text{VIMP}^{(n)}(X_i) = \text{VIMP}(X_i)$$

C) Simulation parameters σ_1^2 and σ_2^2

Derivation

$$\begin{aligned}Var(Z) &= Var(\frac{1}{2}X_1 + \frac{1}{2}X_2 + \epsilon_2) \\ &= \frac{1+c}{2} + \sigma_2^2\end{aligned}$$

$$\begin{aligned}Var(Y) &= Var(X_1 + X_2 + X_3 + \epsilon_1 + \epsilon_2) \\ &= 2c + 3 + \sigma_1^2 + \sigma_2^2\end{aligned}$$

$$\begin{aligned}C(Y,Z) &= \frac{Cov(X_1 + X_2 + X_3 + \epsilon_1 + \epsilon_2, \frac{1}{2}X_1 + \frac{1}{2}X_2 + \epsilon_2)}{\sqrt{Var(Y)\cdot Var(Z)}} \\ &= \frac{\frac{1}{2} + \frac{1}{2}c + \frac{1}{2}c + \frac{1}{2} + \sigma_2^2}{\sqrt{Var(Y)\cdot Var(Z)}} \\ &= \frac{(1+c) + \sigma_2^2}{\sqrt{(\frac{1+c}{2} + \sigma_2^2)\cdot(2c + 3 + \sigma_1^2 + \sigma_2^2)}}\end{aligned}$$

$$\begin{aligned}spC(Y,Z) &= C(Y,\epsilon_2) = C(X_1 + X_2 + X_3 + \epsilon_1 + \epsilon_2, \epsilon_2) \\ &= \frac{\sigma_2^2}{\sqrt{(2c + 3 + \sigma_1^2 + \sigma_2^2)\cdot\sigma_2^2}}\end{aligned}$$

For any given values of $C(Y,Z)$ and $spC(Y,Z)$ and with $c = C(Y,Z)$ the values σ_1^2 and σ_2^2 can be found by grid search.

Results

Table A1 Simulation designs defined by $C(Y,Z)$ and $spC(Y,Z)$ and the corresponding values for σ_1^2 and σ_2^2

$C(Y,Z)$	$spC(Y,Z)$	σ_1^2	σ_2^2
0.3	0	4.04	0.26
0.3	0.1	0.36	28.09
0.3	0.2	1.69	37.21
0.6	0.2	0.36	4.41
0.6	0.4	1.96	6.25
0.9	0.3	0.49	0.01
0.9	0.6	2.89	0.01

D) Additional simulation results

D.a) Simulation Design A: Regression Forest

Table A2 Simulation results (1 000 simulated datasets, each with n patients) showing empirical rejection probabilities of the proposed resampling tests for a regression Random Forest. Correlations within the simulation model are given as the correlation between Y and Z ($C(Y,Z)$) and semipartial correlation between Y and Z ($spC(Y,Z) := spC(Y,Z|X_1,\ldots,X_5)$). The test is here applied to the variable X_4 with $C(Y,X_4) = spC(Y,X_4) = 0$ for all 7 simulation designs.

Simulation design		Rejection prob. of $H_0^{(1)}$			Rejection prob. of $H_0^{(2)}$		
$C(Y,Z)$	$spC(Y,Z)$	n=500	n=1000	n=5000	n=500	n=1000	n=5000
0.3	0	0.004	0.005	0.020	0.027	0.027	0.020
0.3	0.1	0.000	0.002	0.001	0.048	0.057	0.054
0.3	0.2	0.000	0.002	0.050	0.053	0.054	0.050
0.6	0.2	0.000	0.001	0.000	0.044	0.057	0.047
0.6	0.4	0.001	0.001	0.003	0.053	0.053	0.041
0.9	0.3	0.002	0.004	0.005	0.020	0.024	0.022
0.9	0.6	0.003	0.007	0.007	0.023	0.023	0.023

D.b) Additional simulation design C)

Here we introduce another simulation design, which has more covariates and is more complex compared to Simulation Design A. This model contains strong correlations as well as weak correlations between Y and the different X_i and between the X_i themselves respectively. The exact correlations between all variables are shown in Figure A1, where no connection means that an X_i is independent to all other values. The $(X_1, ..., X_{20}, Y)$ vector was simulated as multivariate normally distributed with the given Covariances and $E(X_i) = E(Y) = 0$ and $Var(X_i) = Var(Y) = 1$.

We then applied our test to all variables. The exact rejection rates of this design are shown in Table A3.

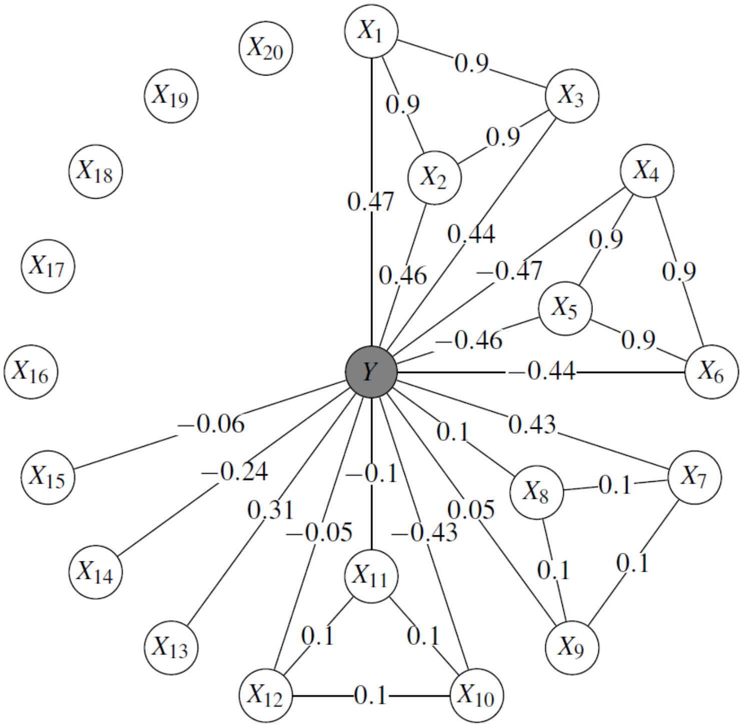

Figure A1 Correlation graph that showws all correlations between Y and the different predictors X_i.

Table A3 Simulation results (1 000 simulated datasets, each with n observations) showing empirical rejection rates for both hypotheses. The true correlations ($C(Y,Z)$) and semi-partial correlations ($spC(Y,Z)$) between Y and the different variables X_i and therefore implicitly the true situation of $H_0^{(1)}$ and $H_0^{(2)}$ are shown in the table as well.

	Variable		Rejection prob. of $H_0^{(1)}$	Rejection prob. of $H_0^{(2)}$
Z=	C(Y,Z)	spC(Y,Z)	n=5000	n=5000
X_1	0.47	0.12	1.000	1.000
X_2	0.46	0.07	1.000	0.999
X_3	0.44	0.00	1.000	0.020
X_4	-0.47	-0.12	1.000	1.000
X_5	-0.46	-0.07	1.000	0.999
X_6	-0.44	0.00	1.000	0.018
X_7	0.43	0.42	0.404	1.000
X_8	0.10	0.06	0.902	0.968
X_9	0.05	0.00	0.253	0.011
X_{10}	-0.43	-0.42	0.409	1.000
X_{11}	-0.10	-0.06	0.894	0.975
X_{12}	-0.05	0.00	0.226	0.009
X_{13}	0.31	0.31	0.008	1.000
X_{14}	-0.24	-0.24	0.013	1.000
X_{15}	-0.06	-0.06	0.016	0.966
X_{16}	0.00	0.00	0.019	0.020
X_{17}	0.00	0.00	0.017	0.017
X_{18}	0.00	0.00	0.015	0.015
X_{19}	0.00	0.00	0.02	0.013
X_{20}	0.00	0.00	0.016	0.024

E) Description of all variables within the application

The variables used in section 5 are given together with the variables' description. The variable names correspond to the names given in figures 2 and A2. The variable names used in the original UNOS dataset are given in brackets.

EPTS Recipient's *Expected Post Transplant Survival*-score. (END_EPTS)[b]

KDPI *Kidney Donor Profile Index* - Score that rates the kidney's quality prior to transplantation. (KDPI)[c]

DIAL_DUR Duration of dialysis prior to transplantation.
(calculated as difference between dialysis start date *(DIAL_DATE)* and transplantation date *(TX_DATE)*).

ETHCAT Recipient's ethnicity category. (ETHCAT)

DIAG Primary diagnosis of recipient's kidney disease. (DIAG_KI)

COLD_ISCH Cold ischemic time of the graft. (COLD_ISCH_KI)

PERIP_VASC Recipient's peripheral vascular disease status. (PERIP_VASC)

BMI Recipient's body mass index. (BMI_CALC)

DIAB Recipient's diabetes status. (DIAB)

AGE Recipient's age. (AGE)

DIAB_DUR Duration of the recipient's diabetes disease.
(calculated as difference between *(AGE)* and *(AGE_DIAB)* that are contained within the raw dataset).

CMV Recipient's CMV status. (CMV_STATUS)

GENDER Recipient's gender. (GENDER)

SERUM_ALB Recipient's total albumin serum. (TOT_SERUM_ALBUM)

[b] https://unos.org/news/in-focus/what-is-epts/

[c] https://unos.org/news/in-focus/what-is-kdpi-a-new-animation-for-patients-explains-the-kidney-donor-profile-index/

MALIG Information about whether the recipient has any malignant disease history prior to transplantation. (MALIG)

DAYSWAIT Total number of days the recipient spent on the waiting list. (DAYSWAIT_CHRON_KI)

HBV Recipient's Hepatitis-B core antibody. (HBV_CORE)

ORG_REC Specification about wether the graft arrived for the transplantation on ice or pump. (ORG_REC_ON)

PROC_TYPE Procedure type of kidney transplantation. (TX_PROCEDUR_TY_KI)

DRMIS HLA-DR mismatch. (DRMIS)

BMIS HLA-B mismatch. (BMIS)

EBV Recipient's epstein barr virus status prior to transplantation. (EBV_SEROSTATUS)

NUM_PREF_TX The number of transplantations the recipient had prior to the actual transplantation. (NUM_PREF_TX)

AB0_MAT Blood-group match level between recipient and donor (ABO_MAT).

AMIS HLA-A mismatch. (AMIS)

AB0 Recipient's blood group. (AB0).

CPRA CPRA value coresponding to transplantation. (END_CPRA_DETAIL)

EGFR Estimated glomerular filtration rate prior to the transplantation. (EGFR_CKDEPI_TRR_PRETX)

F) VIMPS of all variables based on both model descriptions

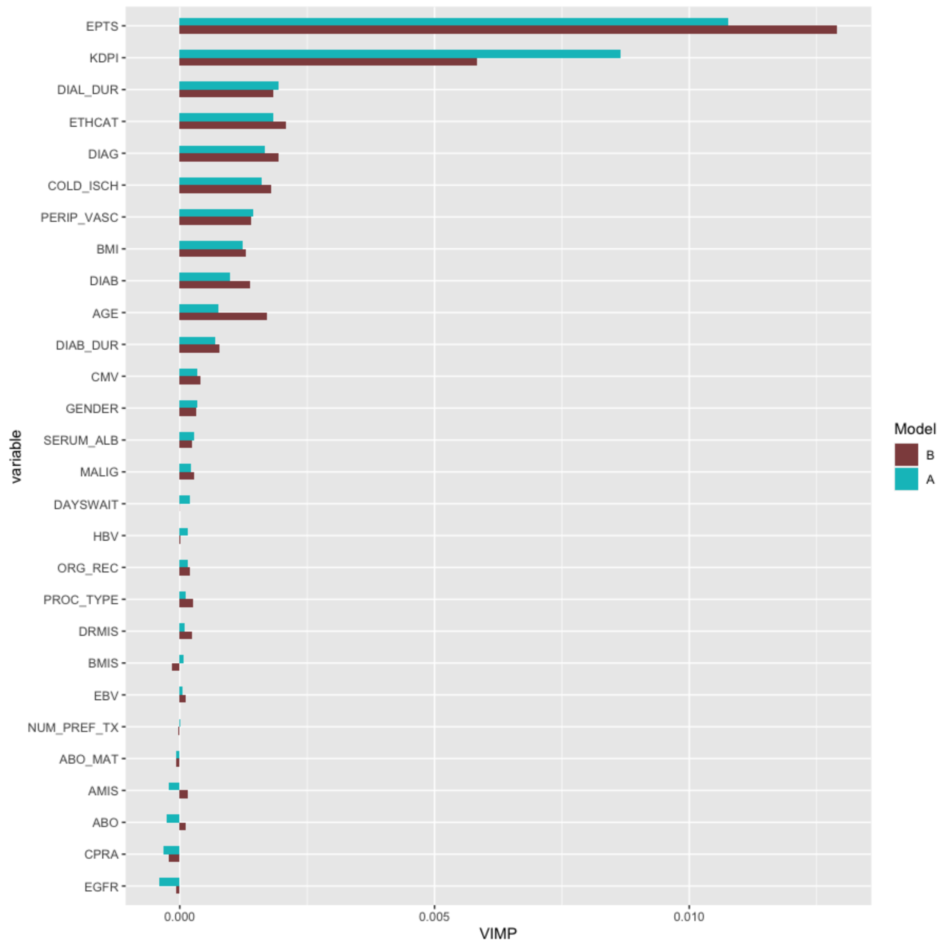

Figure A2 The figure shows the VIMPs of all variables in the Random Survival Forest analysis of UNOS data under model description model$_A$ as well as the corresponding VIMPS under model description model$_B$. Thereby, in model$_B$ KDPI was replaced by its residuals whereas all other variables remained unchanged. Thus, VIMP$_B$(KDPI) refers to residual information and all other VIMPs to original variables' information within the two models.
